# Global Excess Mortality during COVID-19 Pandemic: A Systematic Review and Meta-Analysis

**DOI:** 10.3390/vaccines10101702

**Published:** 2022-10-12

**Authors:** Weijing Shang, Yaping Wang, Jie Yuan, Zirui Guo, Jue Liu, Min Liu

**Affiliations:** School of Public Health, Peking University, Beijing 100191, China

**Keywords:** COVID-19, SARS-CoV-2, excess mortality

## Abstract

Background: Currently, reported COVID-19 deaths are inadequate to assess the impact of the pandemic on global excess mortality. All-cause excess mortality is a WHO-recommended index for assessing the death burden of COVID-19. However, the global excess mortality assessed by this index remains unclear. We aimed to assess the global excess mortality during the COVID-19 pandemic. Methods: We searched PubMed, EMBASE, and Web of Science for studies published in English between 1 January 2020, and 21 May 2022. Cross-sectional and cohort studies that reported data about excess mortality during the pandemic were included. Two researchers independently searched the published studies, extracted data, and assessed quality. The Mantel–Haenszel random-effects method was adopted to estimate pooled risk difference (RD) and their 95% confidence intervals (CIs). Results: A total of 79 countries from twenty studies were included. During the COVID-19 pandemic, of 2,228,109,318 individuals, 17,974,051 all-cause deaths were reported, and 15,498,145 deaths were expected. The pooled global excess mortality was 104.84 (95% CI 85.56–124.13) per 100,000. South America had the highest pooled excess mortality [134.02 (95% CI: 68.24–199.80) per 100,000], while Oceania had the lowest [−32.15 (95% CI: −60.53–−3.77) per 100,000]. Developing countries had higher excess mortality [135.80 (95% CI: 107.83–163.76) per 100,000] than developed countries [68.08 (95% CI: 42.61–93.55) per 100,000]. Lower middle-income countries [133.45 (95% CI: 75.10–191.81) per 100,000] and upper-middle-income countries [149.88 (110.35–189.38) per 100,000] had higher excess mortality than high-income countries [75.54 (95% CI: 53.44–97.64) per 100,000]. Males had higher excess mortality [130.10 (95% CI: 94.15–166.05) per 100,000] than females [102.16 (95% CI: 85.76–118.56) per 100,000]. The population aged ≥ 60 years had the highest excess mortality [781.74 (95% CI: 626.24–937.24) per 100,000]. Conclusions: The pooled global excess mortality was 104.84 deaths per 100,000, and the number of reported all-cause deaths was higher than expected deaths during the global COVID-19 pandemic. In South America, developing and middle-income countries, male populations, and individuals aged ≥ 60 years had a heavier excess mortality burden.

## 1. Introduction

As of 21 May 2022, more than 6.29 million people worldwide died from COVID-19 infection [1]. Global countries have adopted a series of public health measures to curb the coronavirus disease 2019 (COVID-19) pandemic, such as strict lockdown policies and wearing masks [2,3]. These measures cut off the main transmission routes of respiratory infectious diseases such as COVID-19 and influenza and lower their prevalence and mortality [2,3]. Notably, despite the reduction of accidental traffic deaths due to strict lockdown policies [4,5,6,7], these policies may increase the deaths of patients with chronic diseases because they have difficulty receiving timely health care [8,9,10]. Additionally, limited medical resources increase the risk of death among patients with chronic diseases [11]. The deaths from mental depression, suicide, and violence also increased during the pandemic [4,12,13]. Therefore, the COVID-19 pandemic is threatening global health resources and economic and political development.

Mortality statistics are fundamental to the decision-making of public health [14]. However, the categorization of death is inconsistent among countries, health systems, and physicians [3,9]. Importantly, COVID-19 deaths may be underestimated in the early stages of the pandemic because many cases and deaths that should have been attributed to COVID-19 were not detected and identified due to inadequate tests and overloaded health systems, caused by a sudden increase in COVID-19 symptom patients in most countries [15]. Furthermore, influenced by misdiagnosis and pandemic “bias”, indirect deaths during the pandemic are likely to be misclassified as direct deaths of COVID-19, such as deaths caused by resource constraints in health care systems, unnatural causes, or extreme events [9,14,16]. Even before the major reshuffling of death causes due to COVID-19, death certificates were known to be notoriously error-prone. Comorbidities may complicate the assignment of COVID-19 and other illnesses on the death certificate [8,17,18].

Currently, challenges exist in distinguishing death causes induced by COVID-19 or other events. A modeling study indicates that reported COVID-19 deaths are inadequate to assess the impact of the pandemic on excess mortality [14]. Excess mortality is a more comprehensive index to measure the impact of the COVID-19 pandemic on deaths, and it refers to the number of deaths from all causes during the pandemic more than what we would have expected to see under “normal” conditions, including the deaths induced by a lack of medical resources and restrictive intervention during the pandemic [19,20,21]. Moreover, excess mortality can be the reference for assessing COVID-19 deaths because further studies can estimate COVID-19 deaths based on this result by subtracting other causes of death from excess mortality (e.g., heat waves, war, etc.) [22].

At present, various studies from many countries have analyzed excess mortality during the pandemic, whereas global excess mortality is unclear. Most countries, such as the United States, India, and the United Kingdom [23,24,25,26], reported all-cause excess mortality more than the expected level, but it was not in other countries such as Australia and Japan [15,26,27]. Therefore, we conducted this systematic review and meta-analysis study to evaluate global excess mortality and provide evidence regarding the hazard of the pandemic.

## 2. Materials and Methods

### 2.1. Search Strategy

We conducted the meta-analysis following the Preferred Reporting Items for Systematic Reviews and Meta-Analyses (PRISMA) guidelines [28]. This review was registered with PROSPERO (CRD42022334486). Two researchers (WS and ZG) searched published English-language studies from 1 January 2020, to 21 May 2022, through PubMed, EMBASE, and Web of Science. The search terms included (“SARS-CoV-2” or “COVID-19”) and (“Excess Mortality” or “Excess Death” or “Additional Death”). The detailed search strategies are shown in the eMethods in the Supplemental Materials: Text S1. WS and ZG independently reviewed the titles, abstracts, and full texts of articles, and identified additional studies from the reference lists. Disagreements were resolved by discussion with 2 other authors (YW and JY).

The primary outcome to evaluate excess death was excess mortality during the pandemic, defined as the difference between the number of reported all-cause deaths and the expected number of deaths during the pandemic divided by the total population during the same period [29]. The calculation formula was $\mathrm{excess}\;\mathrm{mortality}=\frac{reported\;deaths-expected\;deaths\;}{population}\times 100,000$.

### 2.2. Inclusion and Exclusion Criteria

The inclusion criteria were as follows: (1) observational studies (cross-sectional studies and cohort studies) and (2) studies with extractable data to calculate the excess mortality. We excluded the following studies: (1) duplicates and (2) nonoriginal articles, such as reviews and comments; (3) articles unable to find full text; (4) studies with insufficient data to calculate excess mortality; (5) preprints; (6) overlap studies; (7) non-English studies; (8) nongeneral population studies.

For more than one study in a country, we selected the study that covered the largest population, spanned the longest time period, and performed analyses in different age and sex groups.

### 2.3. Data Extraction

The authors WS and WY independently screened the titles and abstracts, and excluded studies that did not meet the inclusion criteria. Discrepancies were resolved by discussion with the main author (JY). The following data were extracted independently by two authors (WS and WY) from the included studies: first author, publication year, country, study design, the number of reported all-cause deaths, the number of expected all-cause deaths, the number of population, pandemic time and time used to estimate expected deaths. If available, we also extracted the data on the sex and age of reported all-cause deaths, expected all-cause deaths, and the population.

### 2.4. Risk of Bias Assessment

WS and YW independently assessed the risk of bias for each study, which was cross-checked by ZG and JY. Cross-sectional studies were assessed by the Agency for Healthcare Research and Quality (AHRQ) [30] and cohort studies were assessed by the Newcastle–Ottawa scale (Table S1) [31]. Reviewers rated each domain for overall risk of bias as low, moderate, high, or serious/critical (Table S2).

### 2.5. Data Synthesis and Statistical Analysis

We performed a meta-analysis of global excess mortality during the pandemic, and we reported the pooled risk difference (RD) as excess mortality. The Mantel–Haenszel random-effects method [32] was adopted to estimate the pooled risk difference and their 95% confidence intervals (CIs). The lower limit of 95% CI > 0 indicated that the number of reported all-cause deaths was higher than that of expected deaths; the upper limit of 95% CI < 0 indicated that the number of reported all-cause deaths was lower than that of expected deaths; that the 95% CI included 0 suggested no significant difference between reported and expected deaths. The heterogeneity among the studies was estimated using *I*^2^ values. Very low, low, moderate, and high degrees of heterogeneity were defined as *I*^2^ ≤ 25%, 25% to ≤50%, 50% to ≤75%, and ≥75%, respectively [33].

We performed subgroup analyses in continents (Asia vs. Africa vs. Europe vs. North America vs. South America vs. Oceania), country development levels (developing country vs. developed country), World Bank income levels (lower middle-income country vs. upper middle-income country vs. high-income country), age groups (<40 years vs. 40–60 years vs. ≥60 years) and sex (male vs. female). We performed sensitivity analyses by excluding countries with populations less than 1 million. All analyses were performed using Stata software (version 12.0; Stata SE Corporation LP, College Station, TX, USA). A two-sided *p* value < 0.05 was considered statistically significant [34].

## 3. Results

### 3.1. Characteristics of Included Studies

**Figure 1:**
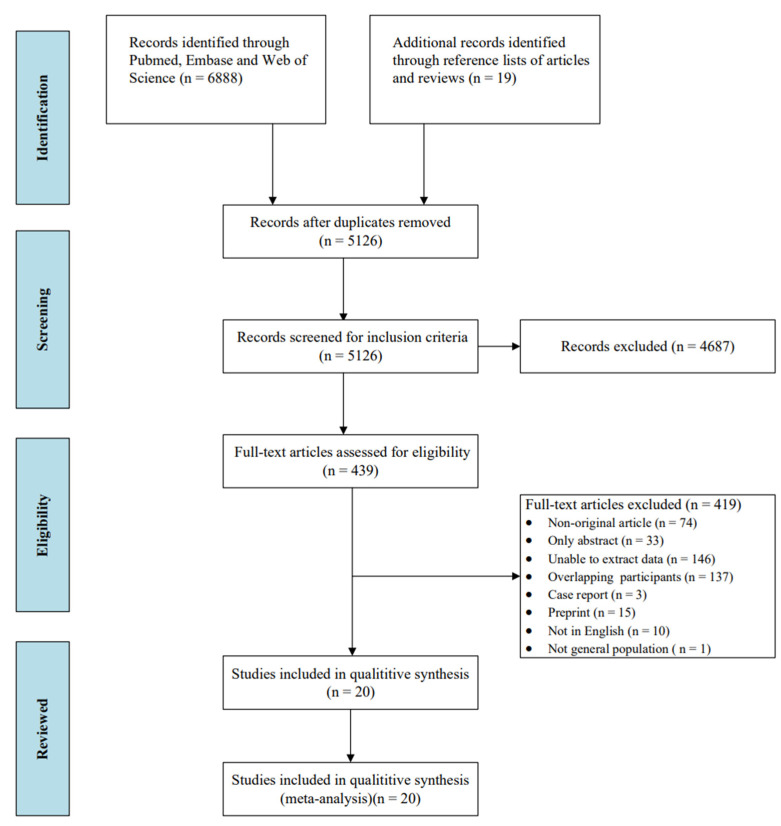
Flowchart of the study selection.

A total of 6907 studies were initially identified through searching the database and the reference list of articles and reviews. Among them, 1781 duplicates and 4687 irrelevant articles were excluded. After exclusion, 439 studies were eligible for full-text review. The final meta-analysis comprised 20 eligible studies (Figure 1), References [15,21,24,25,26,35,36,37,38,39,40,41,42,43,44,45,46,47,48,49] of which 79 countries were included in the study (Table 1).

Among the 20 studies, 2 (10.0%) were cohort studies, and 18 (90.0%) were cross-sectional studies. Nineteen (95.0%) studies were assessed as having a low risk of bias, and one (5.0%) was assessed as having a moderate risk of bias. Among 79 countries, 23 (29.1%) were conducted in Asia, 39 (49.4%) in Europe, 4 (5.1%) in North America, 8 (10.1%) in South America, 3 (3.8%) in Africa, and 2 (2.5%) in Oceania. Forty-three (54.4%) were developing countries, and 36 (45.6%) were developed countries. Nine (11.4%) were lower-middle-income countries, 24 (30.4%) were upper-middle-income countries, and 46 (58.2%) were high-income countries.

### 3.2. Excess Mortality during the COVID-19 Pandemic

**Figure 2:**
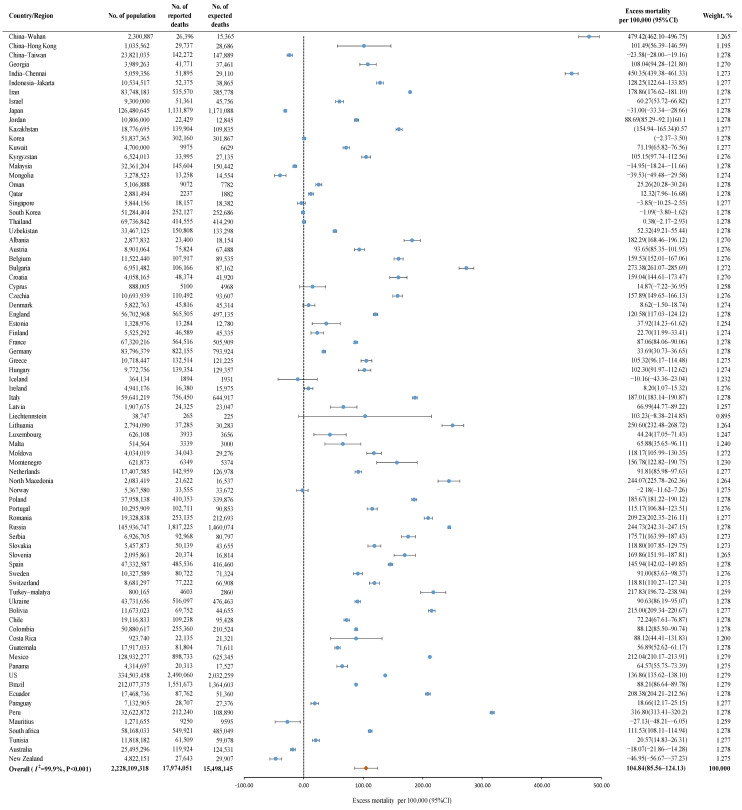
The global excess mortality during the COVID-19 pandemic among 79 countries. Orange dot: the pooled excess mortality; Blue dot: excess mortality in different countries; CI: confidence interval.

A total of 79 countries from 20 studies were included. During the COVID-19 pandemic, of 2,228,109,318 individuals, 17,974,051 all-cause deaths were reported, and 15,498,145 deaths were expected. The pooled global excess mortality was 104.84 (95% CI 85.56–124.13) per 100,000, with high heterogeneity among countries (*I*^2^ = 99.9%) (Figure 2).

**Figure 3:**
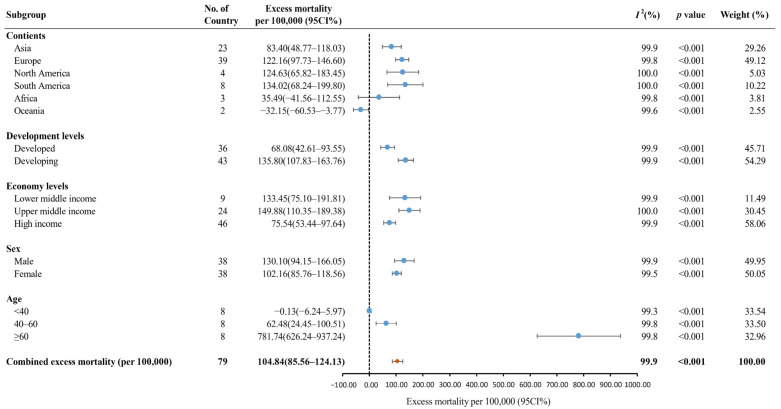
The global excess mortality during the COVID-19 pandemic by subgroup. Orange dot: the pooled excess mortality; Blue dot: excess mortality in different subgroups; CI: confidence interval.

Figure 3 shows the results of the subgroup analysis. South America had the highest pooled excess mortality [134.02 deaths (95% CI: 68.24–199.80) per 100,000], followed by North America [124.63 deaths (95% CI: 65.82–183.45) per 100,000], Europe [122.16 deaths (95% CI: 97.73–146.60) per 100,000], Asia [83.40 deaths (95% CI: 48.77–118.03) per 100,000], Africa [35.49 deaths (95% CI:−41.56–112.55) per 100,000], and Oceania [−32.15 deaths (95% CI:−60.53–−3.77) per 100,000]. The pooled excess mortality was higher in developing countries [135.80 deaths (95% CI: 107.83–163.76) per 100,000] than in developed countries [68.08 deaths (95% CI%: 42.61–93.55) per 100,000]. The pooled excess mortality in lower-middle-income countries [133.45 deaths (95% CI: 75.10–191.81) per 100,000] and upper-middle-income countries [149.88 deaths (110.35 –189.38) per 100,000] was higher than that in high-income countries [75.54 deaths (95% CI: 53.44–97.64) per 100,000]. The pooled excess mortality was higher in males [130.10 deaths (95% CI: 94.15–166.05) per 100,000] than females [102.16 deaths (95% CI: 85.76–118.56) per 100,000]. In eight countries, the population aged ≥ 60 had the highest excess mortality [781.74 deaths (95% CI: 626.24–937.24) per 100,000], followed by the population aged 40–60 [62.48 deaths (95% CI: 24.45–100.51) per 100,000] and aged < 40 [−0.13 deaths (95% CI: −6.24–5.97) per 100,000].

### 3.3. Publication Bias and Sensitivity Analysis

We did not analyze publication bias because the study subject in our study was the country, not the originally published studies. After excluding eight countries with a study population of less than 1 million, the pooled excess mortality [106.99 deaths (95% CI: 86.71–127.27) per 100,000] was similar to the original result. (Figure S1)

## 4. Discussion

In this systematic review and meta-analysis, we found that the pooled excess mortality was 104.84 deaths (95% CI: 85.56–124.13) per 100,000. We found that the excess mortality was higher in South America, North America, Europe, developing countries, lower- or upper-middle-income countries, the male population, and the population aged ≥ 60 years.

To the best of our knowledge, the current study is the first systematic review to evaluate all-cause excess mortality during the pandemic. In this study, the pooled excess mortality was 104.84 per 100,000 globally. Our results are consistent with previous findings (120.30 per 100,000) from COVID-19 Excess Mortality Collaborators, although their findings were derived from a model estimation covering 187 countries/regions [14]. Previous literature reported all-cause excess mortality from January to August 2020 for 22 countries but did not calculate the pooled excess mortality [15]. Similarly, another study calculated all-cause excess mortality during the SARS-CoV-2 pandemic in 67 countries, and no pooled excess mortality data were presented [26]. The coronavirus not only directly kills people but also causes a chain reaction of premature deaths in society. For example, in response to the ongoing epidemic crisis, the Greek public healthcare system ceased most of its regular activities and redirected available resources to COVID-19 treatment and caused excess non-COVID-19 deaths (representing 62% of all-cause excess deaths) during the first 9 months of the epidemic [50]. A similar situation occurred in Italy and England, where 20% and 25% of excess deaths during the first wave of the epidemic could not be directly attributed to COVID-19, respectively [51,52]. Besides, lacking guidelines and personal protective equipment also downsized the clinical activities of primary care centers, which may have increased excess non-COVID-19 deaths during the pandemic [50].

We found that South America had the highest pooled excess mortality, followed by North America, Europe, Asia, Africa, and Oceania, and reported all-cause deaths in Oceania were lower than expected deaths. Our findings are consistent with the COVID-19 Excess Mortality Collaborators’ results [14]. In our study, the top three countries in South America for excess mortality were Ecuador, Bolivia, and Peru, which is consistent with the findings of Karlinsky et al. [3]. In the early stages of the pandemic, the number of deaths increased dramatically in Ecuador due to limited detection capacity and inadequate emergency measures, such as social distancing and wearing masks [49]. In Peru, many factors contributed to all-cause deaths, including coronavirus infection, overloaded health systems, lack of medical services, limited number of ICU beds, and inadequate oxygen supply equipment during the pandemic [9].

North America and Europe were two continents with excess mortality that was only lower than that of South America. Lower mask use, more frequent population mobility, and fewer social distancing mandates may cause high all-cause excess mortality in the United States and parts of European countries [14]. In this study, Africa had low excess mortality, but the prevalence of COVID-19 was severe in sub-Saharan Africa. Thus, we consider that underreporting of deaths or lack of mortality-related surveillance or reporting in some countries may lead to a low rate [14,53,54]. The number of reported all-cause deaths in Oceania was lower than the expected deaths, which is consistent with the findings of previous studies [14,15]. This phenomenon may be related to the following reasons: First, Australia and New Zealand have implemented strict entry-exit screening, timely detection, vaccination and mask-wearing requirements, close contact tracking, and vulnerable group attention during the pandemic [55,56,57,58]. Second, the medical information surveillance system plays an important role in the timely response to public health emergencies in Australia [59]. Third, unique meteorological factors and the Australian government’s influenza vaccination campaign during the pandemic reduced the number of influenza deaths [15]. All the above actions may potentially reduce the number of all-cause deaths in Oceania during the pandemic.

We found that the pooled excess mortality in developing countries was higher than that in developed countries, and middle-income countries had higher excess mortality than high-income countries. The results of the COVID-19 excess mortality collaborators supported that East Asia, Australia, and the high-income Asia-Pacific region had low excess mortality [14], which is similar to our findings. The pandemic has brought shocks to health systems in countries worldwide. Our analysis suggests that developed countries have better quality and more adequate quantities of health care services (e.g., number of intensive care beds, oxygen ventilators, etc.) [60] compared to developing countries. Thus, these advantages might reduce all-cause excess mortality in developed countries. In addition, vaccination is an important protective factor in reducing excess deaths globally, and studies have shown that the number of new deaths per million people decreases over time as vaccine coverage rises [61]. At the beginning of the outbreak, developed countries had better access to the COVID-19 vaccine and higher public accessibility to vaccination, so their vaccine coverage was higher than that of developing countries [62].

In this systematic review, the male population had higher excess mortality than the female population, which is consistent with previous studies [41,63,64,65]. Males with COVID-19 infection have longer courses and worse prognoses than females. In addition, androgens, especially testosterone, are considered a possible risk factor [66]. Populations older than 60 years had higher excess mortality, and several country studies also indicate the same results [42,45,67,68]. We believe that the senior population has lower physical function and immunity compared to younger people, and they are more susceptible to the neo-crown virus during the pandemic [69,70]. Meanwhile, elderly people tend to suffer from one or more chronic diseases, and they are at a higher risk of death due to neo-coronavirus or post-infection complications after unfortunate infections. Moreover, the elderly population is more concerned about the effects of adverse vaccine reactions, and therefore, vaccine hesitancy leads to relatively low vaccination rates in this population. Especially developed countries have a much larger proportion of the elderly population and the excess number of deaths from this population is greater [69,70]. 

Our meta-analysis still has several limitations. First, the number of reported all-cause deaths is real-world data from the mortality surveillance system or death survey in 79 countries. Despite quality control of the data, it is possible that all-cause excess mortality in some countries is underestimated due to delayed or omissive. Because robust vital registration systems do not exist in many parts of the world, the WHO estimated that 40% of global deaths that occurred in 2020 were unregistered [71]. Second, only 10.1% of countries reported the number of all-cause reported deaths and expected deaths by sex and age groups. It is necessary to refine and supplement excess mortality results for sex and age by including more country data in the future. Third, the number of African countries included in this study is small. However, Africa has a severe prevalence of COVID-19 with a potentially high number of excess deaths. Relevant studies in Africa are required to further complement all-cause excess mortality globally.

## 5. Conclusions

In this meta-analysis, the pooled global excess mortality was 104.84 deaths per 100,000, and the number of all-cause reported deaths was higher than expected deaths during the COVID-19 pandemic worldwide. Excess mortality was higher in South America, North America, Europe, developing countries, middle-income countries, the male population, and individuals aged ≥ 60 years. Further research needs to more accurately estimate all-cause excess mortality attributed to the COVID-19 pandemic.

## Figures and Tables

**Table 1:** Characteristics of the studies included in the systematic review and meta-analysis.

First Author, Year	Country	Study Design	No. of Population	No. of Reported Deaths	No. of Expected Deaths	Age Group (Year)	Sex	Continent	Country/Region Development Levels	World Bank Income Levels	COVID-19 Epidemic Period	Time Used to Estimate Expected Deaths (Year)
Liu et al.,2021	China-Wuhan	Cross-sectional	2,300,887	26,396	15,365	/	/	Asia	Developing	Upper Middle	1 January 2020–31 March 2020	2015–2019
Wai et al.,2022	China-Hong kong	Cohort	516,903	16,024	15,827	/	Male	Asia	Developed	High	1 January 2020–31 August 2020	2019
Wai et al., 2022	China-Hong kong	Cohort	518,659	13,713	12,859	/	Female	Asia	Developed	High	1 January 2020–31 August 2020	2019
Sanmarchi et al., 2021	China-Taiwan	Cross-sectional	23,821,035	142,272	147,889	/	/	Asia	Developed	High	26 February 2020–31 December 2020	2018–2019
Sanmarchi et al., 2021	Georgia	Cross-sectional	3,989,263	41,771	37,461	/	/	Asia	Developing	Upper Middle	26 February 2020–31 December 2020	2018–2019
Lewnard et al., 2022	India-Chennai	Cross-sectional	3,057,053	3283	3090	<40	/	Asia	Developing	Lower Middle	1 May 2020–31 August 2020~1 March 2021–30 June 2021	2016–2020
Lewnard et al., 2022	India-Chennai	Cross-sectional	1,421,061	125,00	6950	40–60	/	Asia	Developing	Lower Middle	1 May 2020–31 August 2020~1 March 2021–30 June 2021	2016–2020
Lewnard et al., 2022	India-Chennai	Cross-sectional	581,242	36,130	19,060	≥60	/	Asia	Developing	Lower Middle	1 May 2020–31 August 2020~1 May 2021–30 June 2021	2016–2020
Wijaya et al.,2022	Indonesia-Jakarta	Cross-sectional	5,318,831	30,033	21,842	/	Male	Asia	Developing	Lower Middle	1 June 2020–31 December 2020	2018–2020
Wijaya et al., 2022	Indonesia-Jakarta	Cross-sectional	5,215,686	22,342	17,022	/	Female	Asia	Developing	Lower Middle	1 June 2020–31 December 2020	2018–2020
Safavi-Naini et al., 2022	Iran	Cross-sectional	83,748,183	535,570	385,778	/	/	Asia	Developing	Lower Middle	22 June 2020– 21 March 2021	2013–2019
Peretz et al., 2022	Israel	Cross-sectional	9,300,000	51,361	45,756	/	/	Asia	Developed	High	23 March 2020–28 March 2021	2000–2019
Sanmarchi et al., 2021	Japan	Cross-sectional	126,480,645	1,131,879	1,171,088	/	/	Asia	Developed	High	26 February 2020–31 December 2020	2018–2019
Khader et al., 2021	Jordan	Cross-sectional	5,722,000	13,378	4888	/	Male	Asia	Developing	Upper Middle	1 April 2020–31 December 2020	2016–2019
Khader et al., 2021	Jordan	Cross-sectional	5,084,000	9051	7957	/	Female	Asia	Developing	Upper Middle	1 April 2020–31 December 2020	2016–2019
Sanmarchi et al., 2021	Kazakhstan	Cross-sectional	18,776,695	139,904	109,835	/	/	Asia	Developing	Upper Middle	26 February 2020–31 December 2020	2018–2019
Shin et al., 2021	Korea	Cross-sectional	51,837,365	302,160	301,867	/	/	Asia	Developed	High	1 January 2020–31 December 2020	2010–2019
Alahmad et al., 2021	Kuwait	Cross-sectional	4,700,000	9975	6629	/	/	Asia	Developing	High	1 January 2020–31 December 2020	2015–2019
Sanmarchi et al., 2021	Kyrgyzstan	Cross-sectional	6,524,013	33,995	27,135	/	/	Asia	Developing	Lower Middle	26 February 2020–31 December 2020	2018–2019
Sanmarchi et al., 2021	Malaysia	Cross-sectional	32,361,204	145,604	150,442	/	/	Asia	Developing	Upper Middle	26 February 2020–31 December 2020	2018–2019
Sanmarchi et al., 2021	Mongolia	Cross-sectional	3,278,523	13,258	14,554	/	/	Asia	Developing	Lower Middle	26 February 2020–31 December 2020	2018–2019
Sanmarchi et al., 2021	Oman	Cross-sectional	5,106,888	9072	7782	/	/	Asia	Developing	High	26 February 2020–31 December 2020	2018–2019
Sanmarchi et al., 2021	Qatar	Cross-sectional	2,881,494	2237	1882	/	/	Asia	Developing	High	26 February 2020–31 December 2020	2018–2019
Sanmarchi et al., 2021	Singapore	Cross-sectional	5,844,156	18,157	18,382	/	/	Asia	Developed	High	26 February 2020–31 December 2020	2018–2019
Sanmarchi et al., 2021	South Korea	Cross-sectional	51,284,404	252,127	252,686	/	/	Asia	Developed	High	26 February 2020–31 December 2020	2018–2019
Sanmarchi et al., 2021	Thailand	Cross-sectional	69,736,842	414,555	414,290	/	/	Asia	Developing	Upper Middle	26 February 2020–31 December 2020	2018–2019
Sanmarchi et al., 2021	Uzbekistan	Cross-sectional	33,467,125	150,808	133,298	/	/	Asia	Developing	Lower Middle	26 February 2020–31 December 2020	2018–2019
Rangachev et al., 2022	Cyprus	Cross-sectional	434,471	2707	2635	/	Male	Asia	Developed	High	1 January 2020–31 December 2020	2015–2019
Rangachev et al., 2022	Cyprus	Cross-sectional	453,534	2393	2333	/	Female	Asia	Developed	High	1 January 2020–31 December 2020	2015–2019
Sanmarchi et al., 2021	Mauritius	Cross-sectional	1,271,655	9250	9595	/	/	Africa	Developing	Upper Middle	26 February 2020–31 December 2020	2018–2019
Bradshaw et al., 2021	South Africa	Cross-sectional	58,168,033	549,921	485,049	/	/	Africa	Developing	Upper middle income	1 January 2020–31 December 2020	2018–2019
Sanmarchi et al., 2021	Tunisia	Cross-sectional	11,818,182	61,509	59,078	/	/	Africa	Developing	Lower Middle	26 February 2020–31 December 2020	2018–2019
Sanmarchi et al., 2021	Albania	Cross-sectional	2,877,832	23,400	18,154	/	/	Europe	Developing	Upper Middle	26 February 2020–31 December 2020	2018–2019
Rangachev et al., 2022	Austria	Cross-sectional	4,378,772	37,503	32,975	/	Male	Europe	Developed	High	1 January 2020–31 December 2020	2015–2019
Rangachev et al., 2022	Austria	Cross-sectional	4,522,292	38,321	34,513	/	Female	Europe	Developed	High	1 January 2020–31 December 2020	2015–2019
Rangachev et al., 2022	Belgium	Cross-sectional	5,681,225	52,830	43,967	/	Male	Europe	Developed	High	1 January 2020–31 December 2020	2015–2019
Rangachev et al., 2022	Belgium	Cross-sectional	5,841,215	55,087	45,568	/	Female	Europe	Developed	High	1 January 2020–31 December 2020	2015–2019
Rangachev et al., 2022	Bulgaria	Cross-sectional	3,369,646	56,325	45,372	/	Male	Europe	Developing	Upper Middle	1 January 2020–31 December 2020	2015–2019
Rangachev et al., 2022	Bulgaria	Cross-sectional	3,581,836	49,841	41,790	/	Female	Europe	Developing	Upper Middle	1 January 2020–31 December 2020	2015–2019
Rangachev et al., 2022	Croatia	Cross-sectional	1,971,650	23,907	20,651	/	Male	Europe	Developing	High	1 January 2020–31 December 2020	2015–2019
Rangachev et al., 2022	Croatia	Cross-sectional	2,086,515	24,467	21,269	/	Female	Europe	Developing	High	1 January 2020–31 December 2020	2015–2019
Rangachev et al., 2022	Czechia	Cross-sectional	5,271,996	57,027	47,928	/	Male	Europe	Developed	High	1 January 2020–31 December 2020	2015–2019
Rangachev et al., 2022	Czechia	Cross-sectional	5,421,943	53,465	45,679	/	Female	Europe	Developed	High	1 January 2020–31 December 2020	2015–2019
Rangachev et al., 2022	Denmark	Cross-sectional	2,896,918	23,475	23,184	/	Male	Europe	Developed	High	1 January 2020–31 December 2020	2015–2019
Rangachev et al., 2022	Denmark	Cross-sectional	2,925,845	22,341	22,129	/	Female	Europe	Developed	High	1 January 2020–31 December 2020	2015–2019
Konstantinoudis et al., 2021	England	Cross-sectional	28,051,858	285,683	245,052	/	Male	Europe	Developed	High	1 January 2020–31 December 2020	2015–2019
Konstantinoudis et al., 2021	England	Cross-sectional	28,651,110	279,822	252,083	/	Female	Europe	Developed	High	1 January 2020–31 December 2020	2015–2019
Konstantinoudis et al., 2021	England	Cross-sectional	28,314,021	10,817	12,521	<40	/	Europe	Developed	High	1 January 2020–31 December 2020	2015–2019
Konstantinoudis et al., 2021	England	Cross-sectional	14,728,847	45,084	40,926	40–60	/	Europe	Developed	High	1 January 2020–31 December 2020	2015–2019
Konstantinoudis et al., 2021	England	Cross-sectional	13,660,100	509,604	443,688	≥60	/	Europe	Developed	High	1 January 2020–31 December 2020	2015–2019
Rangachev et al., 2022	Estonia	Cross-sectional	629,277	6266	5992	/	Male	Europe	Developed	High	1 January 2020–31 December 2020	2015–2019
Rangachev et al., 2022	Estonia	Cross-sectional	699,699	7018	6788	/	Female	Europe	Developed	High	1 January 2020–31 December 2020	2015–2019
Rangachev et al., 2022	Finland	Cross-sectional	27,728,262	23,449	22,798	/	Male	Europe	Developed	High	1 January 2020–31 December 2020	2015–2019
Rangachev et al., 2022	Finland	Cross-sectional	2,797,030	23,140	22,537	/	Female	Europe	Developed	High	1 January 2020–31 December 2020	2015–2019
Rangachev et al., 2022	France	Cross-sectional	32,532,669	283,193	251,718	/	Male	Europe	Developed	High	1 January 2020–31 December 2020	2015–2019
Rangachev et al., 2022	France	Cross-sectional	34,787,547	281,323	254,191	/	Female	Europe	Developed	High	1 January 2020–31 December 2020	2015–2019
Sanmarchi et al., 2021	Germany	Cross-sectional	83,796,379	822,155	793,924	/	/	Europe	Developed	High	1 January 2020–31 December 2020	2018–2019
Konstantinoudis et al., 2021	Greece	Cross-sectional	5,215,425	66,856	61,476	/	Male	Europe	Developed	High	1 January 2020–31 December 2020	2015–2019
Konstantinoudis et al., 2021	Greece	Cross-sectional	5,503,022	65,658	59,749	/	Female	Europe	Developed	High	1 January 2020–31 December 2020	2015–2019
Konstantinoudis et al., 2021	Greece	Cross-sectional	4,911,980	1768	1994	<40	/	Europe	Developed	High	1 January 2020–31 December 2020	2015–2019
Konstantinoudis et al., 2021	Greece	Cross-sectional	3,114,996	9051	8750	40–60	/	Europe	Developed	High	1 January 2020–31 December 2020	2015–2019
Konstantinoudis et al., 2021	Greece	Cross-sectional	2,691,471	121,695	110,481	≥60	/	Europe	Developed	High	1 January 2020–31 December 2020	2015–2019
Bogos et al., 2021	Hungary	Cross-sectional	4,393,484	2295	2436	<40	/	Europe	Developing	High	1 January 2020–31 December 2020	2015–2020
Bogos et al., 2021	Hungary	Cross-sectional	2,794,442	14,575	15,191	40–60	/	Europe	Developing	High	1 January 2020–31 December 2020	2015–2021
Bogos et al., 2021	Hungary	Cross-sectional	2,584,830	122,484	112,650	≥60	/	Europe	Developing	High	1 January 2020–31 December 2020	2015–2022
Rangachev et al., 2022	Iceland	Cross-sectional	186,941	980	995	/	Male	Europe	Developed	High	1 January 2020–31 December 2020	2015–2019
Rangachev et al., 2022	Iceland	Cross-sectional	177,193	914	936	/	Female	Europe	Developed	High	1 January 2020–31 December 2020	2015–2019
Achilleos et al., 2021	Ireland	Cross-sectional	2,451,575	8328	8347	/	Male	Europe	Developed	High	1 January 2020–30 August 2020	2015–2019
Achilleos et al., 2021	Ireland	Cross-sectional	2,489,602	8052	7629	/	Female	Europe	Developed	High	1 January 2020–30 August 2020	2015–2019
Konstantinoudis et al., 2021	Italy	Cross-sectional	29,050,086	368,316	308,989	/	Male	Europe	Developed	High	1 January 2020–31 December 2020	2015–2019
Konstantinoudis et al., 2021	Italy	Cross-sectional	30,591,133	388,134	335,928	/	Female	Europe	Developed	High	1 January 2020–31 December 2020	2015–2019
Konstantinoudis et al., 2021	Italy	Cross-sectional	23,536,674	7118	8101	<40	/	Europe	Developed	High	1 January 2020–31 December 2020	2015–2019
Konstantinoudis et al., 2021	Italy	Cross-sectional	18,351,674	42,074	39,743	40–60	/	Europe	Developed	High	1 January 2020–31 December 2020	2015–2019
Konstantinoudis et al., 2021	Italy	Cross-sectional	17,752,871	707,258	597,073	≥60	/	Europe	Developed	High	1 January 2020–31 December 2020	2015–2019
Rangachev et al., 2022	Latvia	Cross-sectional	880,956	11,377	10,840	/	Male	Europe	Developed	High	1 January 2020–31 December 2020	2015–2019
Rangachev et al., 2022	Latvia	Cross-sectional	1,026,719	12,948	12,207	/	Female	Europe	Developed	High	1 January 2020–31 December 2020	2015–2019
Rangachev et al., 2022	Liechtennstein	Cross-sectional	19,215	138	115	/	Male	Europe	Developing	High	1 January 2020–31 December 2020	2015–2019
Rangachev et al., 2022	Liechtennstein	Cross-sectional	19,532	127	110	/	Female	Europe	Developing	High	1 January 2020–31 December 2020	2015–2019
Rangachev et al., 2022	Lithuania	Cross-sectional	1,304,354	18,278	14,455	/	Male	Europe	Developed	High	1 January 2020–31 December 2020	2015–2019
Rangachev et al., 2022	Lithuania	Cross-sectional	1,489,736	19,007	15,828	/	Female	Europe	Developed	High	1 January 2020–31 December 2020	2015–2019
Rangachev et al., 2022	Luxembourg	Cross-sectional	314,964	2022	1850	/	Male	Europe	Developed	High	1 January 2020–31 December 2020	2015–2019
Rangachev et al., 2022	Luxembourg	Cross-sectional	311,144	1911	1806	/	Female	Europe	Developed	High	1 January 2020–31 December 2020	2015–2019
Rangachev et al., 2022	Malta	Cross-sectional	265,762	1709	1575	/	Male	Europe	Developed	High	1 January 2020–31 December 2020	2015–2019
Rangachev et al., 2022	Malta	Cross-sectional	248,802	1630	1425	/	Female	Europe	Developed	High	1 January 2020–31 December 2020	2015–2019
Sanmarchi et al., 2021	Moldova	Cross-sectional	4,034,019	34,043	29,276	/	/	Europe	Developing	Upper Middle	26 February 2020–31 December 2020	2018–2019
Rangachev et al., 2022	Momtenegro	Cross-sectional	307,555	3423	2856	/	Male	Europe	Developing	Upper Middle	1 January 2020–31 December 2020	2015–2019
Rangachev et al., 2022	Momtenegro	Cross-sectional	314,318	2926	2518	/	Female	Europe	Developing	Upper Middle	1 January 2020–31 December 2020	2015–2019
Rangachev et al., 2022	Netherlands	Cross-sectional	8,648,031	71,757	62,423	/	Male	Europe	Developed	High	1 January 2020–31 December 2020	2015–2019
Rangachev et al., 2022	Netherlands	Cross-sectional	8,759,554	71,202	64,555	/	Female	Europe	Developed	High	1 January 2020–31 December 2020	2015–2019
Sanmarchi et al., 2021	North Macedonia	Cross-sectional	2,083,419	21,622	16,537	/	/	Europe	Developing	Upper Middle	1 January 2020–31 December 2020	2018–2019
Rangachev et al., 2022	Norway	Cross-sectional	2,706,562	16,593	16,543	/	Male	Europe	Developed	High	1 January 2020–31 December 2020	2015–2019
Rangachev et al., 2022	Norway	Cross-sectional	2,661,018	16,962	17,129	/	Female	Europe	Developed	High	1 January 2020–31 December 2020	2015–2019
Rangachev et al., 2022	Poland	Cross-sectional	18,373,381	215,400	175,422	/	Male	Europe	Developed	High	1 January 2020–31 December 2020	2015–2019
Rangachev et al., 2022	Poland	Cross-sectional	19,584,757	194,953	164,454	/	Female	Europe	Developed	High	1 January 2020–31 December 2020	2015–2019
Rangachev et al., 2022	Portugal	Cross-sectional	4,859,977	51,086	45,750	/	Male	Europe	Developed	High	1 January 2020–31 December 2020	2015–2019
Rangachev et al., 2022	Portugal	Cross-sectional	5,435,932	51,624	45,103	/	Female	Europe	Developed	High	1 January 2020–31 December 2020	2015–2019
Rangachev et al., 2022	Romania	Cross-sectional	9,460,661	135,274	111,851	/	Male	Europe	Developing	High	1 January 2020–31 December 2020	2015–2019
Rangachev et al., 2022	Romania	Cross-sectional	9,868,177	117,860	100,842	/	Female	Europe	Developing	High	1 January 2020–31 December 2020	2015–2019
Sanmarchi et al., 2021	Russia	Cross-sectional	145,936,747	1,817,225	1,460,074	/	/	Europe	Developing	Upper Middle	26 February 2020–31 December 2020	2018–2019
Rangachev et al., 2022	Serbia	Cross-sectional	3,374,639	48,636	40,923	/	Male	Europe	Developing	Upper Middle	1 January 2020–31 December 2020	2015–2019
Rangachev et al., 2022	Serbia	Cross-sectional	3,552,066	44,332	39,874	/	Female	Europe	Developing	Upper Middle	1 January 2020–31 December 2020	2015–2019
Rangachev et al., 2022	Slovakia	Cross-sectional	2,665,350	25,853	22,517	/	Male	Europe	Developed	High	1 January 2020–31 December 2020	2015–2019
Rangachev et al., 2022	Slovakia	Cross-sectional	2,792,523	24,286	21,138	/	Female	Europe	Developed	High	1 January 2020–31 December 2020	2015–2019
Rangachev et al., 2022	Slovenia	Cross-sectional	1,051,066	9972	8314	/	Male	Europe	Developed	High	1 January 2020–31 December 2020	2015–2019
Rangachev et al., 2022	Slovenia	Cross-sectional	1,044,795	10,402	8500	/	Female	Europe	Developed	High	1 January 2020–31 December 2020	2015–2019
Konstantinoudis et al., 2021	Spain	Cross-sectional	23,199,257	247,003	211,135	/	Male	Europe	Developed	High	1 January 2020–31 December 2020	2015–2019
Konstantinoudis et al., 2021	Spain	Cross-sectional	24,133,330	238,533	205,325	/	Female	Europe	Developed	High	1 January 2020–31 December 2020	2015–2019
Konstantinoudis et al., 2021	Spain	Cross-sectional	20,276,614	6305	6433	<40	/	Europe	Developed	High	1 January 2020–31 December 2020	2015–2019
Konstantinoudis et al., 2021	Spain	Cross-sectional	14,869,360	34,577	33,741	40–60	/	Europe	Developed	High	1 January 2020–31 December 2020	2015–2019
Konstantinoudis et al., 2021	Spain	Cross-sectional	12,186,613	444,654	376,286	≥60	/	Europe	Developed	High	1 January 2020–31 December 2020	2015–2019
Rangachev et al., 2022	Sweden	Cross-sectional	5,195,814	40,286	34,921	/	Male	Europe	Developed	High	1 January 2020–31 December 2020	2015–2019
Rangachev et al., 2022	Sweden	Cross-sectional	5,131,775	40,436	36,403	/	Female	Europe	Developed	High	1 January 2020–31 December 2020	2015–2019
Konstantinoudis et al., 2021	Switzerland	Cross-sectional	4,309,104	38,099	32,311	/	Male	Europe	Developed	High	1 January 2020–31 December 2020	2015–2019
Konstantinoudis et al., 2021	Switzerland	Cross-sectional	4,372,193	39,123	34,597	/	Female	Europe	Developed	High	1 January 2020–31 December 2020	2015–2019
Konstantinoudis et al., 2021	Switzerland	Cross-sectional	4,020,006	1377	1324	<40	/	Europe	Developed	High	1 January 2020–31 December 2020	2015–2019
Konstantinoudis et al., 2021	Switzerland	Cross-sectional	2,499,892	4531	4653	40–60	/	Europe	Developed	High	1 January 2020–31 December 2020	2015–2019
Konstantinoudis et al., 2021	Switzerland	Cross-sectional	2,161,399	71,314	60,931	≥60	/	Europe	Developed	High	1 January 2020–31 December 2020	2015–2019
Aytemur et al., 2021	Turkey-malatya	Cross-sectional	800,165	4603	2860	/	/	Europe	Developing	Upper Middle	1 January 2020–31 December 2020	2016–2019
Sanmarchi et al., 2021	Ukraine	Cross-sectional	43,731,656	516,097	476,463	/	/	Europe	Developing	Lower Middle	26 February 2020–31 December 2020	2018–2019
Sanmarchi et al., 2021	Costa Rica	Cross-sectional	923,740	22,135	21,321	/	/	North America	Developing	Upper Middle	26 February 2020–31 December 2020	2018–2019
Sanmarchi et al., 2021	Guatemala	Cross-sectional	17,917,033	81,804	71,611	/	/	North America	Developing	Upper Middle	26 February 2020–31 December 2020	2018–2019
Sanmarchi et al., 2021	Mexico	Cross-sectional	12,8932,277	898,733	625,345	/	/	North America	Developing	Upper Middle	26 February 2020–31 December 2020	2018–2019
Jacobson et al., 2021	US	Cohort	165,036,419	1,305,641	1,043,584	/	Male	North America	Developed	High	1 March 2020–28 November 2020	2015–2019
Jacobson et al., 2021	US	Cohort	169,467,039	1,184,419	988,675	/	Female	North America	Developed	High	1 March 2020–28 November 2020	2015–2019
Sanmarchi et al., 2021	Australia	Cross-sectional	25,495,296	119,924	124,531	/	/	Oceania	Developed	High	26 February 2020–31 December 2020	2018–2019
Sanmarchi et al., 2021	New Zealand	Cross-sectional	4,822,151	27,643	29,907	/	/	Oceania	Developed	High	26 February 2020–31 December 2020	2018–2019
Sanmarchi et al., 2021	Bolivia	Cross-sectional	11,673,023	69,752	44,655	/	/	South America	Developing	Lower Middle	26 February 2020–31 December 2020	2018–2019
Sanmarchi et al., 2021	Chile	Cross-sectional	19,116,833	109,238	95,428	/	/	South America	Developing	High	26 February 2020–31 December 2020	2018–2019
Sanmarchi et al., 2021	Colombia	Cross-sectional	50,880,617	255,360	210,524	/	/	South America	Developing	Upper Middle	26 February 2020–31 December 2020	2018–2019
Sanmarchi et al., 2021	Panama	Cross-sectional	4,314,697	20,313	17,527	/	/	South America	Developing	High	26 February 2020–31 December 2020	2018–2019
Cue’ llar et al., 2022	Ecuador	Cross-sectional	17,468,736	87,762	51,360	/	/	South America	Developing	Upper Middle	1 January 2020–26 September 2020	2015–2019
Sanmarchi et al., 2021	Paraguay	Cross-sectional	7,132,905	28,707	27,376	/	/	South America	Developing	Upper Middle	26 February 2020–31 December 2020	2018–2019
Ramírez-Soto et al., 2022	Peru	Cross-sectional	16,198,980	127,000	58,392	/	Male	South America	Developing	Upper Middle	1 January 2020–31 December 2020	2017–2019
Ramírez-Soto et al., 2022	Peru	Cross-sectional	16,423,892	85,240	50,498	/	Female	South America	Developing	Upper Middle	1 January 2020–31 December 2020	2017–2019
Santos et al., 2021	Brazil	Cross-sectional	104,546,709	870,431	752,451	/	Male	South America	Developing	Upper Middle	29 December 2019–2 January 2021	2015–2019
Santos et al., 2021	Brazil	Cross-sectional	107,530,666	681,242	612,152	/	Female	South America	Developing	Upper Middle	29 December 2019–2 January 2021	2015–2019
Santos et al., 2021	Brazil	Cross-sectional	129,649,264	179,254	165,879	<40	/	South America	Developing	Upper Middle	29 December 2019–2 January 2021	2015–2019
Santos et al., 2021	Brazil	Cross-sectional	53,137,449	289,529	242,597	40–60	/	South America	Developing	Upper Middle	29 December 2019–2 January 2021	2015–2019
Santos et al., 2021	Brazil	Cross-sectional	29,290,662	1,082,890	956,127	≥60	/	South America	Developing	Upper Middle	29 December 2019–2 January 2021	2015–2019

## Data Availability

Data can be obtained by contacting the corresponding author.

## Supplementary Materials

The following supporting information can be downloaded at: https://www.mdpi.com/article/10.3390/vaccines10101702/s1, Text S1: Search strategy; Table S1: Risk of bias of all included cross-sectional studies using the Agency for Healthcare Research and Quality scale (AHRQ-Tool) (*n* = 18); Table S2: Risk of bias of all included cohort studies using the Newcastle-Ottawa quality assessment scale (*n* = 2); Figure S1: Sensitive analysis of excess mortality among 71 countries/regions.
