# Supplementary material for: Global Excess Mortality during COVID-19 Pandemic: A Systematic Review and Meta-Analysis
Source: Vaccines (Basel). 2022 Oct 12;10(10):1702. doi: 10.3390/vaccines10101702 (PMC9607451; doi:10.3390/vaccines10101702)
Supplement: Supplementary file 1 [file vaccines-10-01702-s001.zip › vaccines-1943329-supplementary.pdf]

Supplemental materialse

Methods Search Strategies Text S1

PubMed Advance Search

#1 Search: (COVID-19[MeSH Terms]) OR (SARS-CoV-2[MeSH Terms])

#2 ((((Severe Acute Respiratory Syndrome Coronavirus 2) OR (coronavirus disease 2019)) OR (sars-cov*)) OR (SARS Coronavirus*)) OR (covid*)

#3 ((COVID-19[MeSH Terms]) OR (SARS-CoV-2[MeSH Terms])) OR (((((Severe Acute Respiratory Syndrome Coronavirus 2) OR (coronavirus disease 2019)) OR (sars-cov*)) OR (SARS Coronavirus*)) OR (covid*))

#4 Search: ((additional death*) OR (excess death*)) OR (excess mortalit*)

#5 (((COVID-19[MeSH Terms]) OR (SARS-CoV-2[MeSH Terms])) OR (((((Severe Acute Respiratory Syndrome Coronavirus 2) OR (coronavirus disease 2019)) OR (sars-cov*)) OR (SARS Coronavirus*)) OR (covid*))) AND (((additional death*) OR (excess death*)) OR (excess mortalit*))

#6 ("2020/01/01"[Date - Publication] : "2022/05/21"[Date - Publication])

#7 ((((COVID-19[MeSH Terms]) OR (SARS-CoV-2[MeSH Terms])) OR (((((Severe Acute Respiratory Syndrome Coronavirus 2) OR (coronavirus disease 2019)) OR (sars-cov*)) OR (SARS Coronavirus*)) OR (covid*))) AND (((additional death*) OR (excess death*)) OR (excess mortalit*))) AND (("2020/01/01"[Date - Publication] : "2022/05/21"[Date - Publication]))

Embase Advance Search

#1 **'severe acute respiratory syndrome coronavirus 2'**/exp

#2 **'coronavirus disease 2019'**/exp

#3 **'sars cov*'** OR **'sars coronavirus*'** OR **covid***

#4 #1 OR #2 OR #3

#5 **'excess mortality'**/exp

#6 **'additional death*'** OR **'excess death*'** OR **'excess mortalit*'**

#7 #5 OR #6

#8 #4 AND #7

#9 #4 AND #7 AND [embase]/lim AND [01-01-2020]/sd NOT [22-05-2022]/sd

Web of Science Advance Search

TS= (SARS-CoV* OR covid* OR Severe acute respiratory syndrome coronavirus 2 OR coronavirus disease 2019 OR SARS Coronavirus*) AND TS= (additional death* OR excess death* OR excess mortalit*)

Country/Region	No. of Population	No. of reported deaths	No. of expected deaths	Excess mortality per 100,000 (95%CI)	Weight, %
China–Wuhan	2,300,887	26,396	15,365	479.42(462.10–496.75)	1.399
China–Hong Kong	1,035,562	29,737	28,686	101.49(56.39–146.59)	1.321
China–Taiwan	23,821,035	142,272	147,889	−23.58(−28.00–−19.16)	1.413
Georgia	3,989,263	41,771	37,461	108.04(94.28–121.80)	1.405
India–Chennai	5,059,356	51,895	29,110	450.35(439.38–461.33)	1.408
Indonesia–Jakarta	10,534,517	52,375	38,865	128.25(122.64–133.85)	1.412
Iran	83,748,183	535,570	385,778	178.86(176.62–181.10)	1.414
Israel	9,300,000	51,361	45,756	60.27(53.72–66.82)	1.412
Japan	126,480,645	1,131,879	1,171,088	−31.00(−33.34–−28.66)	1.414
Jordan	10,806,000	22,429	12,845	88.69(85.29–92.10)	1.413
Kazakhstan	18,776,695	139,904	109,835	160.14(154.94–165.34)	1.413
Korea	51,837,365	302,160	301,867	0.57(−2.37–3.50)	1.413
Kuwait	4,700,000	9975	6629	71.19(65.82–76.56)	1.412
Kyrgyzstan	6,524,013	33,995	27,135	105.15(97.74–112.56)	1.411
Malaysia	32,361,204	145,604	150,442	−14.95(−18.24–−11.66)	1.413
Mongolia	3,278,523	13,258	14,554	−39.53(−49.48–−29.58)	1.409
Oman	5,106,888	9072	7782	25.26(20.28–30.24)	1.413
Qatar	2,881,494	2237	1882	12.32(7.96–16.68)	1.413
Singapore	5,844,156	18,157	18,382	−3.85(−10.25–2.55)	1.412
South Korea	51,284,404	252,127	252,686	−1.09(−3.80–1.62)	1.413
Thailand	69,736,842	414,555	414,290	0.38(−2.17–2.93)	1.414
Uzbekistan	33,467,125	150,808	133,298	52.32 (49.21–55.44)	1.413
Albania	2,877,832	23,400	18,154	182.29 (168.46–196.12)	1.405
Austria	8,901,064	75,824	67,488	93.65 (85.35–101.95)	1.410
Belgium	11,522,440	107,917	89,535	159.53 (152.01–167.06)	1.411
Bulgaria	6,951,482	106,166	87,162	273.38 (261.07–285.69)	1.407
Croatia	4,058,165	48,374	41,920	159.04 (144.61–173.47)	1.404
Czechia	10,693,939	110,492	93,607	157.89 (149.65–166.13)	1.411
Denmark	5,822,763	45,816	45,314	8.62 (−1.50–18.74)	1.409
England	56,702,968	565,505	497,135	120.58 (117.03–124.12)	1.413
Estonia	1,328,976	13,284	12,780	37.92 (14.23–61.62)	1.387
Finland	5,525,292	46,589	45,335	22.70(11.99–33.41)	1.408
France	67,320,216	564,516	505,909	87.06(84.06–90.06)	1.413
Germany	83,796,379	822,155	793,924	33.69(30.73–36.65)	1.413
Greece	10,718,447	132,514	121,225	105.32 (96.17–114.48)	1.410
Hungary	9,772,756	139,354	129,357	102.30 (91.97–112.62)	1.409
Ireland	4,941,176	16,380	15,975	8.20(1.07–15.32)	1.411
Italy	59,641,219	756,450	644,917	187.01(183.14–190.87)	1.413
Latvia	1,907,675	24,325	23,047	66.99(44.77–89.22)	1.390
Lithuania	2,794,090	37,285	30,283	250.60(232.48–268.72)	1.398
Moldova	4,034,019	34,043	29,276	118.17(105.99–130.35)	1.407
Netherlands	17,407,585	142,959	126,978	91.81(85.98–97.63)	1.412
North Macedonia	2,083,419	21,622	16,537	244.07 (225.78–262.36)	1.398
Norway	5,367,580	33,555	33,672	−2.18 (−11.62–7.26)	1.410
Poland	37,958,138	410,353	339,876	185.67 (181.22–190.12)	1.413
Portugal	10,295,909	102,711	90,853	115.17 (106.84–123.51)	1.410
Romania	19,328,838	253,135	212,693	209.23 (202.35–216.11)	1.412
Russia	145,936,747	1,817,225	1,460,074	244.73 (242.31–247.15)	1.414
Serbia	6,926,705	92,968	80,797	175.71 (163.99–187.43)	1.407
Slovakia	5,457,873	50,139	43,655	118.80 (107.85–129.75)	1.408
Slovenia	2,095,861	20,374	16,814	169.86 (151.91–187.81)	1.398
Spain	47,332,587	485,536	416,460	145.94 (142.02–149.85)	1.413
Sweden	10,327,589	80,722	71,324	91.00 (83.63–98.37)	1.411
Switzerland	8,681,297	77,222	66,908	118.81 (110.27–127.34)	1.410
Ukraine	43,731,656	516,097	476,463	90.63 (86.19–95.07)	1.413
Bolivia	11,673,023	69,752	44,655	215.00(209.34–220.67)	1.412
Chile	19,116,833	109,238	95,428	72.24(67.61–76.87)	1.413
Colombia	50,880,617	255,360	210,524	88.12(85.50–90.74)	1.414
Guatemala	17,917,033	81,804	71,611	56.89(52.62–61.17)	1.413
Mexico	128,932,277	898,733	625,345	212.04(210.17–213.91)	1.414
Panama	4,314,697	20,313	17,527	64.57(55.75–73.39)	1.410
US	334,503,458	2,490,060	2,032,259	136.86 (135.62–138.1)	1.414
Brazil	212,077,375	1,551,673	1,364,603	88.21 (86.64–89.78)	1.414
Ecuador	17,468,736	87,762	51,360	208.38 (204.21–212.56)	1.413
Paraguay	7,132,905	28,707	27,376	18.66 (12.17–25.15)	1.412
Peru	32,622,872	212,240	108,890	316.8 (313.41–320.2)	1.413
Mauritius	1,271,655	9250	9595	−27.13 (−48.21–−6.05)	1.393
South africa	58,168,033	549,921	485,049	111.53 (108.11–114.94)	1.413
Tunisia	11,818,182	61,509	59,078	20.57 (14.83–26.31)	1.412
Australia	25,495,296	119,924	124,531	−18.07 (−21.86–−14.28)	1.413
New Zealand	4,822,151	27,643	29,907	−46.95 (−56.67–−37.23)	1.409
**Overall (I^2=99.9%, P<0.001)**	**2,223,331,982**	**17,926,433**	**15,454,810**	**106.99(86.71–127.27)**	**100.000**

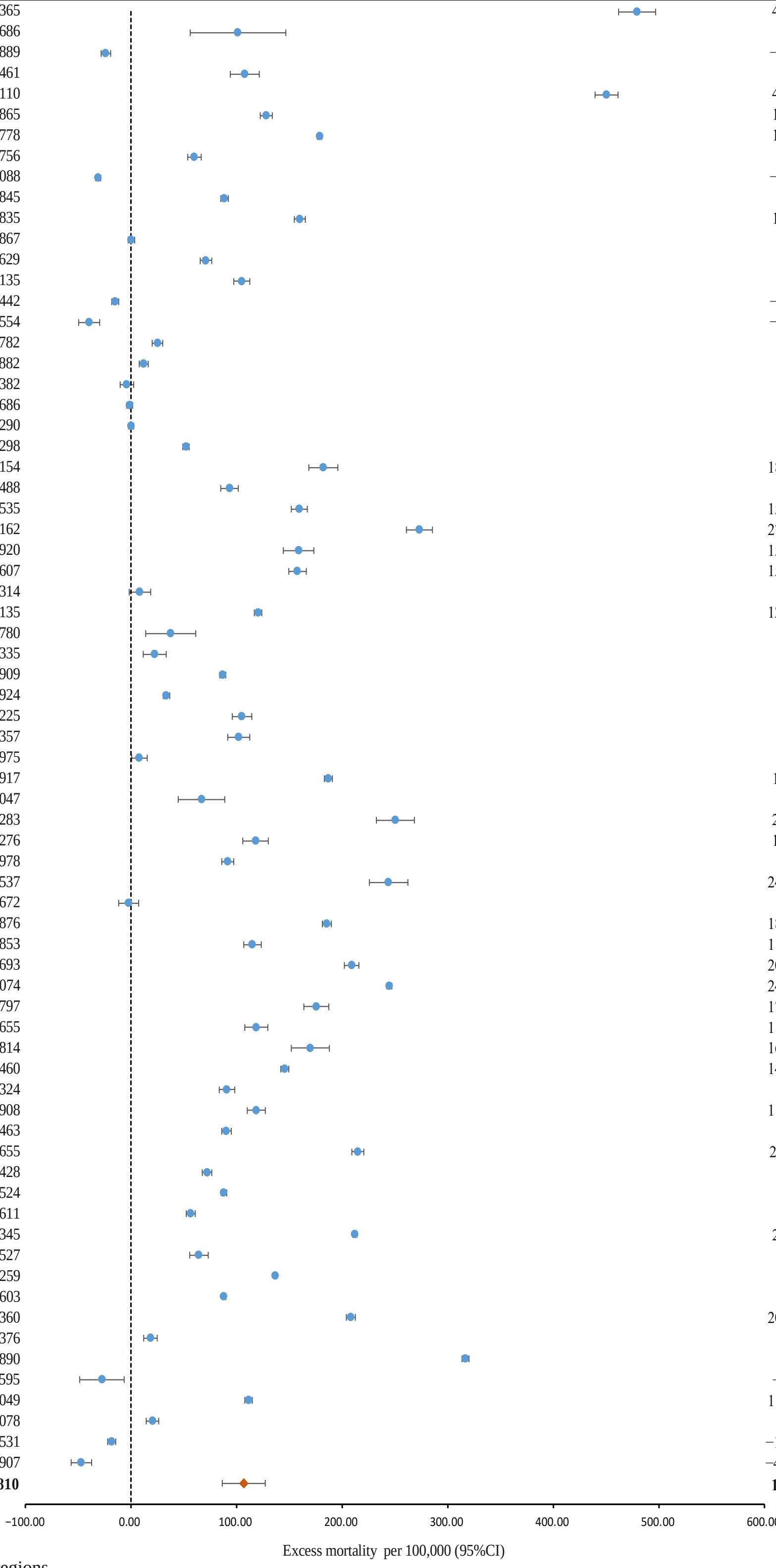

Figure S1 Sensitive analysis of excess mortality among 71 countries/regions
Orange dot: the pooled excess mortality; Blue dot: excess mortality in different countries; CI: confidence interval.

Table S1. Risk of bias of all included cross-sectional studies using the Agency for Healthcare Research and Quality scale (AHRQ-Tool) (n=18)

Author	1) Define the source of information	2) List definition for expected and reported deaths	3) Indicate time period used for identifying death	4) Indicate whether or not subjects were consecutive if not population-based	5) Indicate if evaluators of subjective components of study were masked to other aspects of the status of the participants	6) Describe any assessments undertaken for quality assurance purposes (at least 3 years data)	7) Explain any deaths exclusions from analysis	8) Describe how confounding was assessed (gender, age, etc.)	9) If applicable, explain how missing data were handled in the analysis	10) Summarize completeness of death data collection	11) Clarify reported time and recorded time of deaths and the time lag of deaths for which incomplete data or follow-up was obtained	Total scores	Article quality
Santos et al	yes	yes	yes	yes	yes	yes	yes	yes	unclear	yes	no	9	High
Bogos et al	yes	yes	yes	yes	yes	yes	yes	yes	yes	yes	no	10	High
Alahmad et al	yes	yes	yes	yes	yes	yes	yes	no	yes	yes	no	9	High
Bradshaw et al	yes	yes	yes	yes	yes	no	yes	yes	yes	yes	yes	10	High
Achilleos et al	yes	yes	yes	yes	yes	yes	yes	no	yes	yes	yes	10	High
Aytemur et al	yes	yes	yes	yes	yes	no	yes	no	no	yes	no	7	Moderate
Shin et al	yes	yes	yes	yes	yes	yes	yes	yes	unclear	yes	no	9	High
Ramírez-Soto et al	yes	yes	yes	yes	yes	yes	yes	yes	yes	yes	no	10	High
Safavi-Naini et al	yes	yes	yes	yes	yes	yes	yes	yes	unclear	yes	no	9	High
Rangachev et al	yes	yes	yes	yes	yes	yes	yes	yes	yes	yes	no	10	High
Wijaya et al	yes	yes	yes	yes	yes	yes	yes	yes	no	yes	no	9	High
Peretz et al	yes	yes	yes	yes	yes	yes	yes	yes	unclear	yes	yes	10	Hhigh
Sanmarchi et al	yes	yes	yes	yes	yes	yes	yes	no	yes	yes	no	9	Hhigh
Konstantinoudis et al	yes	yes	yes	yes	yes	yes	yes	yes	unclear	yes	unclear	9	High
Lewnard et al	yes	yes	yes	yes	yes	yes	yes	yes	unclear	yes	no	9	High
Liu et al	yes	yes	yes	yes	yes	yes	yes	yes	unclear	yes	yes	10	High
Khader et al	yes	yes	yes	yes	yes	yes	yes	yes	unclear	yes	no	9	High

Cue´ llar et al	yes	yes	yes	yes	yes	yes	yes	yes	unclear	yes	no	9	High

Note: low quality = 0–3; moderate quality = 4–7; high quality = 8–11

Table S2. Risk of bias of all included cohort studies using the Newcastle-Ottawa quality assessment scale (n=2)

Author	Selection				Comparability	Outcome			Total	Risk level
	Representativeness of the Exposed Cohort	Selection of the Non-Exposed Cohort	Ascertainment of Exposure	Demonstration That Outcome of Interest Was Not Present at Start of Study	Comparability of Cohorts on the Basis of the Design or Analysis	Assessment of Outcome	Was Follow-Up Long Enough for Outcomes to Occur	Adequacy of Follow Up of Cohorts (28days at least)		
Wai et al	1	1	1	0	2	1	1	1	8	Low
Jacobson et al	1	1	1	1	2	1	1	0	8	Low

Note: low (≥7 scores), moderate (5–6 scores), and high risk of bias (≤4 scores)
